# Supplementary material for: Overcoming resolution limits with quantum sensing
Source: Nat Commun. 2019 Nov 1;10:4992. doi: 10.1038/s41467-019-12817-y (PMC6825202; doi:10.1038/s41467-019-12817-y)
Supplement: Supplementary file 1 — Supplementary Information [file 41467_2019_12817_MOESM1_ESM.pdf]

Overcoming resolution limits with quantum sensing-Supplementary material

T. Gefen,[1] A. Rotem,[1] and A. Retzker[1]

[1] *Racah Institute of Physics, The Hebrew University of Jerusalem, Jerusalem 91904, Givat Ram, Israel*

(Dated: September 25, 2019)

Supplementary Note I. Conditions for quantum resolution

The following claim is stated in the main text: Given $\rho\left(\omega_{\mathrm{r}}\right)$ such that $\frac{d\rho}{d\omega_{\mathrm{r}}}=0$ (as $\omega_{\mathrm{r}}\to 0$), then $I_{\mathrm{r}}\left(\omega_{r}\to 0\right)>0$ if and only if one of the eigenvalues of ρ goes as ω_{r}^{k} for $1<k\leq 2$, or equivalently if and only if $\frac{d\sqrt{\rho}}{d\omega_{\mathrm{r}}}\neq 0$. The optimal measurement basis converges to an eigenbasis of ρ as $\omega_{\mathrm{r}}\to 0$. We showed in the main text that the first condition (at least one of the eigenvalues $\sim\omega_{\mathrm{r}}^{1<k\leq 2}$) is sufficient and necessary.

First, let us clarify one point: $\frac{dp_j}{d\omega_{\mathrm{r}}}$ is defined as the derivative of the j-th eigenvalue (at $\omega_{\mathrm{r}}=0$), note that it equals the derivative of the probability of the j-th eigenstate (at $\omega_{\mathrm{r}}=0$), to see this:

$$\frac{d}{d\omega_{\mathrm{r}}}\langle\psi_j|\rho|\psi_j\rangle|_{\omega_{\mathrm{r}}=0}=\langle\psi_j\left(0\right)|\frac{d\rho}{d\omega_{\mathrm{r}}}|\psi_j\left(0\right)\rangle+\langle\frac{d\psi_j}{d\omega_{\mathrm{r}}}|\rho\left(0\right)|\psi_j\left(0\right)\rangle+\langle\psi_j\left(0\right)|\rho\left(0\right)|\frac{d\psi_j}{d\omega_{\mathrm{r}}}\rangle=\langle\psi_j\left(0\right)|\frac{d\rho}{d\omega_{\mathrm{r}}}|\psi_j\left(0\right)\rangle, \tag{1}$$

where $|\psi_j\left(\omega_{\mathrm{r}}\right)\rangle$ is the j-th eigenstate of $\rho\left(\omega_{\mathrm{r}}\right)$. The second equality is due to: $\langle\frac{d\psi_j}{d\omega_{\mathrm{r}}}|\psi_j\rangle+\langle\psi_j|\frac{d\psi_j}{d\omega_{\mathrm{r}}}\rangle=0$. Therefore the eigenbasis of ρ attains the QFI $\left(\sum_j\frac{\left(\frac{dp_j}{d\omega_{\mathrm{r}}}\right)^2}{p_j}\right)$.

An alternative way to see that the eigenbasis is an optimal measurement basis proceeds as follows:

$$\frac{d\rho}{d\omega_{\mathrm{r}}}=0\Rightarrow L\rho+\rho L=0, \tag{2}$$

where L is the symmetric logarithmic derivative operator (its eigenbasis is the optimal measurement basis [1]) . Note that the fact that ρ,L anticommute impliess that $L\rho=\rho L=0$, because given that $|l\rangle$ is an eigenstate of L with an eigenvalue $l\neq 0$, then:

$$\langle l|\left(L\rho+\rho L\right)|l\rangle=\left(l+l^*\right)\langle l|\rho|l\rangle=0\Rightarrow\langle l|\rho|l\rangle=0\Rightarrow\rho|l\rangle=0. \tag{3}$$

So taking an eigenbasis of L: $\{|l_1\rangle,...,|l_n\rangle\}$ with eigenvalues $l_1,...,l_n$. It can be seen that $\forall i\ \rho L|l_i\rangle=0$ and thus $L\rho=\rho L=0$. Therefore they have a common eigenbasis and it attains the QFI.

We next show that the second condition is sufficient and necessary. First, one can see directly that the two conditions are equivalent. Using the same notations as in the main text, we can simply find $\frac{d\sqrt{\rho}}{d\omega_{\mathrm{r}}}$:

$$\frac{d\sqrt{\rho}}{d\omega_{\mathrm{r}}}=\sum_j\frac{\frac{dp_j}{d\omega_{\mathrm{r}}}}{2\sqrt{p_j}}|j\rangle\langle j|+i\sum_{j,k}\left(\sqrt{p_j}-\sqrt{p_k}\right)h_{k,j}|k\rangle\langle j|. \tag{4}$$

Since $\frac{d\rho}{d\omega_{\mathrm{r}}}=0$ then $\left(p_j-p_k\right)h_{k,j}=0$ $(\forall k,j)$ therefore $\left(\sqrt{p_j}-\sqrt{p_k}\right)h_{k,j}=0$. While $\frac{\frac{dp_j}{d\omega_{\mathrm{r}}}}{2\sqrt{p_j}}\neq 0$ if and only if $p_j\sim\omega_{\mathrm{r}}^{k}$ $\left(1<k\leq 2\right)$. Therefore these conditions are equivalent.

In fact the more general statement is: for any $\rho\left(\theta\right)$ the QFI (about θ) vanishes if and only if $\frac{d\sqrt{\rho}}{d\theta}=0$.

This fact is a simple coclusion of the following claim (which we prove):

Claim: The QFI ($\mathscr{F}$) about θ satisfies:

$$2\,\mathrm{trace}\left[\left(\frac{d\sqrt{\rho}}{d\theta}\right)^2\right]\leq\mathscr{F}\leq 4\,\mathrm{trace}\left[\left(\frac{d\sqrt{\rho}}{d\theta}\right)^2\right]. \tag{5}$$

Proof: using supplementary equation 4 we get:

$$\mathrm{trace}\left[\left(\frac{d\sqrt{\rho}}{d\theta}\right)^2\right]=\sum_{j,k}\left(\frac{d\sqrt{\rho}}{d\theta}\right)_{j,k}\left(\frac{d\sqrt{\rho}}{d\theta}\right)_{k,j}=\sum_j\frac{\left(\frac{dp_j}{d\theta}\right)^2}{4p_j}+\sum_{j,k}\left(\sqrt{p_j}-\sqrt{p_k}\right)^2|h_{j,k}|^2. \tag{6}$$

Recall that $\mathscr{F}$ reads:

$$\mathscr{F}=\sum_j\frac{\left(\frac{dp_j}{d\omega_{\mathrm{r}}}\right)^2}{p_j}+2\sum_{j,k}\frac{\left(p_j-p_k\right)^2}{p_j+p_k}|h_{kj}|^2 \tag{7}$$

Now observe that:

$$\frac{(p_j-p_k)^2}{p_j+p_k} = \frac{\left(\sqrt{p_j}-\sqrt{p_k}\right)^2\left(\sqrt{p_j}+\sqrt{p_k}\right)^2}{p_j+p_k} = \left(\sqrt{p_j}-\sqrt{p_k}\right)^2\left[1+\frac{2\sqrt{p_j}\sqrt{p_k}}{p_j+p_k}\right]. \tag{8}$$

Therefore:

$$\left(\sqrt{p_j}-\sqrt{p_k}\right)^2 \leq \frac{(p_j-p_k)^2}{p_j+p_k} \leq 2\left(\sqrt{p_j}-\sqrt{p_k}\right)^2. \tag{9}$$

So on one hand:

$$4\,\mathrm{trace}\left[\left(\frac{d\sqrt{\rho}}{d\theta}\right)^2\right] = \sum_j \frac{\left(\frac{dp_j}{d\theta}\right)^2}{p_j} + 4\sum_{j,k}\left(\sqrt{p_j}-\sqrt{p_k}\right)^2 |h_{j,k}|^2 \geq \sum_j \frac{\left(\frac{dp_j}{d\omega_{\mathrm{r}}}\right)^2}{p_j} + 2\sum_{j,k}\frac{(p_j-p_k)^2}{p_j+p_k}|h_{kj}|^2 = \mathscr{F}, \tag{10}$$

and on the other hand:

$$2\mathrm{trace}\left[\left(\frac{d\sqrt{\rho}}{d\theta}\right)^2\right] = \sum_j \frac{\left(\frac{dp_j}{d\theta}\right)^2}{2p_j} + 2\sum_{j,k}\left(\sqrt{p_j}-\sqrt{p_k}\right)^2 |h_{j,k}|^2 \leq \mathscr{F}. \tag{11}$$

Combining the last two inequalities we get the desired inequality: $2\,\mathrm{trace}\left[\left(\frac{d\sqrt{\rho}}{d\theta}\right)^2\right] \leq \mathscr{F} \leq 4\,\mathrm{trace}\left[\left(\frac{d\sqrt{\rho}}{d\theta}\right)^2\right]$.

Note that the lower bound is saturated for pure states (where only the quantum state is changed) and the upper bound for cases in which only the eigenvalues are changed.

Conclusion: the QFI vanishes if and only if $\frac{d\sqrt{\rho}}{d\theta}$ vanishes.

The proof is immediate: $\left(\frac{d\sqrt{\rho}}{d\theta}\right)^2$ is a positive semidefinite, Hermitian operator. Therefore the trace vanishes if and only if $\left(\frac{d\sqrt{\rho}}{d\theta}\right)^2$ vanishes, that vanishes if and only if $\frac{d\sqrt{\rho}}{d\theta}$ vanishes.

Supplementary Note II. Conditions for superresolution: multivariable case

We wish to prove the condition for a non-singular QFI given that $\left(\frac{\partial\rho}{\partial\theta_i}\right)_{i=1}^n$ are linear dependent (with dimension $k<n$). Let us set the stage for the statement. We can choose the parameters $(\theta_i)_{i=1}^n$ such that $\left(\frac{\partial\rho}{\partial\theta_i}\right)_{i=1}^k$ are linear independent, and $\frac{\partial\rho}{\partial\theta_{k+1}} = ... = \frac{\partial\rho}{\partial\theta_n} = 0$.

Definition: the classical FI matrix of ρ is the FI matrix according to the eigenvalues of ρ (namely the FI matrix achieved when measuring in the eigenbasis of ρ).

The claim is that the QFI matrix is non-singular if and only if the classical FI matrix about the subset $\{\theta_i\}_{i=k+1}^n$ is non-singular.

To prove this claim we use some facts in quantum and classical estimation theory. Recall that the QFI matrix (denoted as $\mathscr{F}$) reads:

$$\mathscr{F}_{m,l} = 2\sum_{i,j}\frac{\left(\frac{\partial\rho}{\partial\theta_m}\right)_{i,j}\left(\frac{\partial\rho}{\partial\theta_l}\right)_{j,i}}{(p_i+p_j)}, \tag{12}$$

where p_j is the j-th eigenvalue of ρ, and the matrix elements are in the eigenbasis of ρ. inserting supplementary equation 4 , we get that:

$$\mathscr{F}_{m,l} = \sum_j \frac{\left(\frac{\partial p_j}{\partial\theta_m}\right)\left(\frac{\partial p_j}{\partial\theta_l}\right)}{p_j} + \sum_{i,j}\frac{(p_i-p_j)^2}{p_i+p_j}\left(h^m_{i,j}h^l_{j,i} + h^m_{j,i}h^l_{i,j}\right), \tag{13}$$

where h^m is the Hermitian operator that corresponds to $\frac{\partial}{\partial\theta_m}$. Note that just like in the single-variable case, the first term is the information that we gain from the change in the eigenvalues and the second term is the information that we gain from the change

in the eigenvectors. The first term is thus the classical FI matrix (defined earlier), and is denoted from now on as C. The second term can be thought of as the quantum part of the QFI, and is denoted from now on as Q. For convenience let us split C and Q into blocks according to $\{\theta_i\}_{i=1}^{k}$ and $\{\theta_i\}_{i=k+1}^{n}$:

$$C=\begin{pmatrix} C^{11} & C^{12} \\ C^{21} & C^{22} \end{pmatrix},\ Q=\begin{pmatrix} Q^{11} & Q^{12} \\ Q^{21} & Q^{22} \end{pmatrix}, \tag{14}$$

where C^{11} is the classical FI about $\{\theta_i\}_{i=1}^{k}$, C^{22} is the classical FI about $\{\theta_i\}_{i=k+1}^{n}$ (and analogously for Q).

Claim 1: Given that $\frac{\partial\rho}{\partial\theta_{k+1}}=...=\frac{\partial\rho}{\partial\theta_n}=0$, then $Q^{22}=0$, $Q^{12}=Q^{21}=0$.

proof: Just like in the single-variable case, $\frac{\partial\rho}{\partial\theta_m}=0$ implies $(\forall i,j)\ (p_i-p_j)\,h_{i,j}^m=0$ and therefore $(\forall l)\ \frac{(p_i-p_j)^2}{p_i+p_j}h_{i,j}^m h_{j,i}^l=0$ (because $\frac{(p_i-p_j)^2}{p_i+p_j}|h_{i,j}^m|\leq(p_i-p_j)\,|h_{i,j}^m|\to 0$). This implies that Q^{22},Q^{12},Q^{21} vanish.

Claim 2: Given that C^{22} is singular, then C is singular, and vectors that nullify C^{22} nullify also C.

proof: Recall that a (classical) FI matrix is defined as:

$$\begin{aligned} I_{m,l} &= \sum_i \frac{\left(\frac{\partial p_i}{\partial\theta_m}\right)\left(\frac{\partial p}{\partial\theta_l}\right)}{p_i} = 4\sum_i\left(\frac{\partial\sqrt{p_i}}{\partial\theta_m}\right)\left(\frac{\partial\sqrt{p_i}}{\partial\theta_l}\right) \\ &= \langle\frac{\partial\sqrt{p}}{\partial\theta_m},\frac{\partial\sqrt{p}}{\partial\theta_l}\rangle. \end{aligned} \tag{15}$$

So it is an inner product matrix between the vectors $\left\{\frac{\partial\sqrt{p}}{\partial\theta_m}\right\}_{m=1}^{n}$. Hence it is regular if and only if these vectors are linear independent, and the null-space is all the linear combinations of these vectors that vanish. Therefore, if C^{22} is singular then $\left\{\frac{\partial\sqrt{p}}{\partial\theta_m}\right\}_{m=k+1}^{n}$ is linear dependent which implies that $\left\{\frac{\partial\sqrt{p}}{\partial\theta_m}\right\}_{m=1}^{n}$ is linear dependent, and the null-space of C^{22} is a subspace of the null-space of C.

This immediately leads to the desired conclusion:

Conclusion: Given that $\left(\frac{\partial\rho}{\partial\theta_i}\right)_{i=1}^{k}$ are linear independent and $\frac{\partial\rho}{\partial\theta_{k+1}}=...=\frac{\partial\rho}{\partial\theta_n}=0$ (the problematic parameters), then the QFI matrix is regular if and only if C^{22} (the classical FI about the problematic parameters) is regular.

Proof: We first show that if C^{22} is singular then the QFI is singular. C^{22} is singular and is thus nullified by a vector $\boldsymbol{\alpha}$. From claim 2, this $\boldsymbol{\alpha}$ nullifies also C, and from claim 1 it nullifies also Q. Therefore the QFI matrix is nullified by $\boldsymbol{\alpha}$ and is thus singular.

We now show that if the QFI is singular then C^{22} is singular. Given that the QFI is nullified by $\boldsymbol{\alpha}$, then $2\sum_{i,j}\frac{|\boldsymbol{\alpha}\cdot(\boldsymbol{\partial}_\theta\boldsymbol{\rho})_{i,j}|^2}{(p_i+p_j)}=0.$ and therefore $\boldsymbol{\alpha}\cdot(\boldsymbol{\partial}_\theta\boldsymbol{\rho})=0$. This means that $\alpha_1=...=\alpha_k=0$, namely this vector is a linear combination of only the problematic parameters and thus $C^{22}\boldsymbol{\alpha}=0$. Hence C^{22} is singular. □

As it is mentioned in a footnote, one can formulate an equivalent condition. Recall that in the single variable case the QFI is positive $\Leftrightarrow\frac{d\sqrt{\rho}}{d\theta}\neq 0$. An immediate conclusion of this is that in the multivariable case the QFI matrix is non-singular $\Leftrightarrow$ $\left\{\frac{\partial\sqrt{\rho}}{\partial\theta_i}\right\}_{i=1}^{n}$ are linear independent: the QFI matrix is singular $\Leftrightarrow$ there exist a parameter y, a linear combination of $(\theta_i)_i$, such that the (single-variable) QFI about y vanishes $\Leftrightarrow\frac{d\sqrt{\rho}}{dy}=0\Leftrightarrow\left\{\frac{\partial\sqrt{\rho}}{\partial\theta_i}\right\}_{i=1}^{n}$ are linear dependent.

Supplementary Note III. Relation to quantum superresolution in imaging

In this part we revisit the recent superresolution scheme proposed in [2] and show that it is a special case of the criterion presented in the main text. In the imaging problem one has two close incoherent optical sources, located in x_1,x_2, and the goal is to determine the number of sources (two or one) and estimate their positions. The probe in this problem is the radiation emitted from the sources (detected by a measurement device). In the far-field limit, all terms higher than single photon terms can be neglected, so that the state of the radiation reads:

$$\rho=(1-\varepsilon)\,|\text{vac}\rangle\langle\text{vac}|+\varepsilon\rho_1+O\left(\varepsilon^2\right),\ \text{where}\ \rho_1=\frac{1}{2}\left(|\psi_1\rangle\langle\psi_1|+|\psi_2\rangle\langle\psi_2|\right), \tag{16}$$

and $|\psi_j\rangle=\int\psi_j(x)\,|1,x\rangle\,dx$ ($|1,x\rangle$ is the state of one photon in position x). $|\psi_j(x)\rangle$ is the photonic wave function corresponding to the j-th source, we consider the symmetric case in which $\psi_j(x)=\psi(x-x_j)$ where x_j is the position of the j-th source.

We can now define the parameters $\theta_1 = \frac{1}{2}(x_1 + x_2)$ (the centroid, equivalent to ω_s in our case), and $\theta_2 = x_1 - x_2$ (the distance between sources, equivalent to ω_r in our case). Replacing $x_1 \longleftrightarrow x_2$ leads to $\psi_1(x) \longleftrightarrow \psi_2(x)$ which does not change ρ. Hence in this case, ρ is symmetric with respect to the parameter θ_2 namely $\rho(\theta_2) = \rho(-\theta_2)$, and thus:

$$\frac{\partial \rho}{\partial \theta_2} \to 0,\ \theta_2 \to 0. \tag{17}$$

Therefore ρ suffers from a vanishing distinguishability. According to the criterion in the main text, the optimal measurement basis would converge to an eigenbasis of ρ and a finite FI about θ_2 can be achieved if and only if one of the eigenvalues $\to 0$ as θ_2^2. Observe that the eigenstates of ρ (in the subspace of one photon states) are $|\psi_1\rangle \pm \frac{\langle\psi_2|\psi_1\rangle}{|\langle\psi_2|\psi_1\rangle|}|\psi_2\rangle$ with eigenvalues $\frac{\varepsilon}{2}(1 \pm |\langle\psi_2|\psi_1\rangle|)$ respectively. Therefore the condition is satisfied given that $1 - |\langle\psi_1|\psi_2\rangle| \sim \theta_2^2$ as $\theta_2 \to 0$, which is the case for a wide variety of $\psi(x)$ (e.g. Gaussian, sinc functions and many more). Hence the superresolution method in this case, as proposed in [2] and according to the criterion in the main text, is to measure whether the one photon state is in $|\psi(x)\rangle$. The probability of not being in this state goes as θ_2^2 and thus a finite FI is achieved in the limit of $\theta_2 \to 0$.

Note that given a symmetric pure state (unnormalized): $|\psi_1\rangle + |\psi_2\rangle$ resolution cannot be achieved (due to purity) just like the resolution limit in spectroscopy given a coherent symmetric signal. The state $\frac{1}{2}(|\psi_1\rangle\langle\psi_1| + |\psi_2\rangle\langle\psi_2|)$ is the ensemble average of states with random phases: $|\psi_1\rangle + e^{i\phi}|\psi_2\rangle$, which is similar to averaging over many realizations of random phase signals that we make in spectroscopy.

Supplementary Note IV. Resolution limitations in quantum spectroscopy

It is shown in the main text that given the following Hamiltonian (identical quadratures):

$$H = \left[\sum_i A\cos(\omega_i t) + B\sin(\omega_i t)\right]\sigma_z = \left[\sum_i \Omega\sin(\omega_i t + \varphi)\right]\sigma_z, \tag{18}$$

($\Omega = \sqrt{A^2 + B^2}, \varphi = \arctan\left(\frac{B}{A}\right)$), then:

$$\frac{\partial|\psi_t\rangle}{\partial\omega_\mathrm{r}} = 0 \Rightarrow I_\mathrm{r} = 0\ (\omega_\mathrm{r} = 0). \tag{19}$$

Note that a similar limitation appears also in the case of identical phases but different amplitudes:

$$H = \sum_i \Omega_i \sin(\omega_i t + \varphi)\sigma_z. \tag{20}$$

Of course, $\frac{\partial H}{\partial\omega_\mathrm{r}} \neq 0$, however neither ω_s nor ω_r can be efficiently estimated: To see this observe that:

$$\forall t\ \Omega_2 \frac{\partial H}{\partial\omega_1} = \Omega_1 \frac{\partial H}{\partial\omega_2}\ (\omega_\mathrm{r} = 0). \tag{21}$$

So we can define $\omega_- = \frac{1}{\sqrt{\Omega_1^2+\Omega_2^2}}(\Omega_2\omega_1 - \Omega_1\omega_2)$, $\omega_+ = \frac{1}{\sqrt{\Omega_1^2+\Omega_2^2}}(\Omega_1\omega_1 + \Omega_2\omega_2)$, and then supplementary equation 21 implies that $\forall t\ \frac{\partial H}{\partial\omega_-} = 0\ (\omega_\mathrm{r} = 0)$, and thus $\Delta\omega_\mathrm{r} \to \infty$.

Now consider the most general case: amplitudes and phases are not necessarily identical:

$$H = \sum_i \Omega_i \sin(\omega_i t + \varphi_i)\sigma_z. \tag{22}$$

Given $\omega_\mathrm{r} t \ll 1$, the Hamiltonian reads:

$$H = \sigma_z\left[\sum_i \Omega_i \sin(\omega_\mathrm{s} t + \varphi_i) + \omega_\mathrm{r} t \sum_i \Omega_i \cos(\omega_\mathrm{s} t + \varphi_i) + \mathscr{O}\left(\omega_\mathrm{r}^2 t^2\right)\right], \tag{23}$$

so neglecting the $(\omega_\mathrm{r} t)^2$ terms the Hamiltonian can be written as:

$$H \approx [a\sin(\omega_\mathrm{s} t + \alpha) + b\omega_\mathrm{r} t\sin(\omega_\mathrm{s} t + \beta)]\sigma_z, \tag{24}$$

where $a\sin(\omega_s t+\alpha)=\sum_i \Omega_i \sin(\omega_s t+\varphi_i)$, and $b\sin(\omega_s t+\beta)=\sum_i \Omega_i \cos(\omega_s t+\varphi_i)$. It would be more convenient then to work with the parameters $\omega_r, \omega_s, a, b, \alpha, \beta$ (instead of $\omega_1, \omega_2, \Omega_1, \Omega_2, \phi_1, \phi_2$). Supplementary eq. 24 immediately implies that the Hamiltonian suffers from a degeneracy, i.e. it depends only on 5 parameters: $a, \alpha, \omega_s, b\omega_r, \beta$. Namely we cannot get information on b, ω_r separately, but only on $b\omega_r$. A different way to phrase this is that $-\omega_r \frac{\partial H}{\partial \omega_r}+b\frac{\partial H}{\partial b}=0$,hence there exists a parameter g such that $\forall t$ $\frac{\partial H}{\partial g}=0$. More elaborately we can see explicitly that $\operatorname{span}\left(\{\nabla f(t)\}_t\right)$ is of dimension ≤ 5 (where $H=f(t)\,\sigma_z$):

$$\nabla f(t)=\begin{pmatrix}\frac{\partial f}{\partial \omega_s}\\ \frac{\partial f}{\partial \omega_r}\\ \frac{\partial f}{\partial a}\\ \frac{\partial f}{\partial b}\\ \frac{\partial f}{\partial \alpha}\\ \frac{\partial f}{\partial \beta}\end{pmatrix}=\begin{pmatrix} at\cos(\omega_s t+\alpha)+b\omega_r t^2\cos(\omega_s t+\beta)\\ bt\sin(\omega_s t+\beta)\\ \sin(\omega_s t+\alpha)\\ \omega_r t\sin(\omega_s t+\beta)\\ a\cos(\omega_s t+\alpha)\\ b\omega_r t\cos(\omega_r t+\beta)\end{pmatrix}$$

$$=t\cos(\omega_s t)\begin{pmatrix} a\cos(\alpha)\\ b\sin(\beta)\\ 0\\ \omega_r\sin(\beta)\\ 0\\ b\omega_r\cos(\beta)\end{pmatrix}+t\sin(\omega_s t)\begin{pmatrix} -a\sin(\alpha)\\ b\cos(\beta)\\ 0\\ \omega_r\cos(\beta)\\ 0\\ -b\omega_r\sin(\beta)\end{pmatrix}+\cos(\omega_s t)\begin{pmatrix}0\\0\\ \sin(\alpha)\\ 0\\ a\cos(\alpha)\\ 0\end{pmatrix}+\sin(\omega_s t)\begin{pmatrix}0\\0\\ \cos(\alpha)\\ 0\\ -a\sin(\alpha)\\ 0\end{pmatrix}+b\omega_r t^2\begin{pmatrix}1\\0\\0\\0\\0\\0\end{pmatrix}. \tag{25}$$

Hence for $\omega_r \neq 0$, the dimension is 5. Note that for $\omega_r=0$ the dimension is 4 (as $\frac{\partial H}{\partial b}=0$, $\frac{\partial H}{\partial \beta}=0$). Therefore in this case as well $\Delta\omega_r \to \infty$.

Supplementary Note V. Effective Hamiltonian: Conditions for a non vanishing FI

The exact expression of the accumulated phase for a single frequency signal (given in the methods section) reads:

$$\phi=A\sin(\delta t)\frac{\tan\left(\omega\frac{\tau}{2}\right)}{\omega}+B\left(1-\cos(\delta t)\right)\frac{\tan\left(\omega\frac{\tau}{2}\right)}{\omega}. \tag{26}$$

Based on this expression, we want to understand what the conditions are on δ (or equivalently τ) to nullify ϕ for every A, B. Clearly, whenever $\omega\tau \neq n\pi$ and $\delta t=2\pi k$ (n, k are integers) $\phi=0$. Note that the condition $\delta t=2\pi k$ implies that $\omega t=\pi m$ (k, m are integers, because the total time is an integer multiple of $\frac{\pi}{\omega+\delta}$). In addition, whenever $\omega\tau=2\pi n$ we have that $\tan\left(\omega\frac{\tau}{2}\right)=0$ and thus $\phi=0$. It is simple to understand this case. The signal completes an integer number of cycles between two consecutive pulses and thus the accumulated phase vanishes. Note that for $\omega\tau=(2n+1)\pi$, it is not possible to nullify ϕ for every A, B. Therefore the condition $\delta t=2\pi k$ is not valid here. In this case: $\phi=A\left(\frac{2}{\pi}\right)\frac{t}{2n+1}$, therefore $A\neq 0 \Rightarrow \phi \neq 0$. For a two frequency signal the accumulated phase is:

$$H=\sum_i\left[A_i\sin(\omega_i t)+B_i\cos(\omega_i t)\right]h(t)\,\sigma_z. \tag{27}$$

In order to get a non vanishing I_r when $\omega_r=0$ we have to nullify ϕ (at $\omega_r=0$). As shown above we need to either take $\delta_s t=2\pi k$ or take $\omega_s\tau=2\pi n$. For the first possibility, given that $\omega_r t \ll 1$, the accumulated phase reads:

$$\phi=\sum_i\frac{\tan\left(\omega_i\frac{\tau}{2}\right)}{\omega_i}\left[A_i\sin(\delta_i t)+B_i\left(1-\cos(\delta_i t)\right)\right], \tag{28}$$

and thus a finite I_r is obtained:

$$I_r=8\sigma^2\frac{\tan\left(\omega_s\frac{\tau}{2}\right)^2}{\omega_s^2}t^2. \tag{29}$$

Note that this expression is not valid for $\omega_s\tau=(2n+1)\pi$, as for these values $\phi\neq 0 \Rightarrow I_r=0$. For the second possibility ($\omega_s\tau=2\pi n$): Note that this option has an overlap with the first one, in that if $\frac{t}{\tau}$ is even then δt is an integer multiplication of 2π. However it can be seen that in this case $\phi\sim\omega_r^2 \Rightarrow p\sim\omega_r^4$ and thus I_r vanishes. If $\frac{t}{\tau}$ is odd (and $\omega_s\tau=2\pi n$) then:

$\phi \approx \frac{(B_1-B_2)\tau^2}{2\pi n}\omega_{\rm r} \Rightarrow I_{\rm r} = \frac{8\sigma^2\tau^4}{(2\pi)^2 n^2}$. Hence a finite $I_{\rm r}$ is achieved but it is much lower than with the first option, as it independent of t, and thus does not grow with t. Therefore we dismiss this option and only keep the first one, in which $\delta_s t = 2\pi k\ (\omega_{\rm s}\tau \neq n\pi)$, and the FI is given in supplementary equation 29.

Naturally we would like to confirm that the optimal detuning (or τ) is $\delta_s = \pm 2\pi/t$. To see this, let us first examine how close $\omega_{\rm s}\tau$ can approach π (while requiring $\delta_s t = 2\pi k \neq 0$):

$$\omega\tau = (\omega+\delta)\tau - \delta\tau = \pi - \delta\tau. \tag{30}$$

Hence the closest it can approach π is by $\delta\tau$. Since the minimal possible δ is $\frac{2\pi}{t}$,we cannot get closer to π than $\frac{2\pi\tau}{t} = \frac{2\pi}{N}$ (where N is the number of pulses). Therefore if the closest we can get to π is $\frac{2\pi}{N} \approx \frac{2\pi^2}{\omega t}$, then the closest we can get to 3π is $\approx \frac{6\pi^2}{\omega t}$, and so on. Therefore the optimal $I_{\rm r}$ is achieved with $\delta_s = \pm\frac{2\pi}{t}$ and reads:

$$I_{\rm r} = 8\sigma^2 \frac{\tan\left(\frac{\pi}{2\left(1\pm\frac{2\pi}{\omega_{\rm s}t}\right)}\right)^2}{\omega_{\rm s}^2} t^2 \approx \frac{8}{\pi^4}\sigma^2 t^4. \tag{31}$$

It is well established that for frequency estimation problems the optimal scaling of the FI is $\sim \Omega^2 t^4$[3–8], where Ω stands for the amplitude of the signal, and this is exactly the scaling that this method achieves. The optimality of this method is discussed in supplementary note VI.

Supplementary Note VI. Optimality analysis

We presented control methods for which $I_{\rm r} \neq 0$, and found the optimal one out of a set of possible controls. However a valid question is whether this method is optimal out of all possible control strategies. This question is left open; however, we can find an upper bound of $I_{\rm r}$ (which is not tight). Given a Hamiltonian:

$$H = \sum_i \left(A_i \cos(\omega_i t) + B_i \sin(\omega_i t)\right)\sigma_Z \tag{32}$$

The optimal $I_{\rm r}$ (FI about $\omega_{\rm r}$) is given by (according to supplementary ref. [3]):

$$I_{\rm r} = 4\left[\int \left|\frac{\partial H}{\partial \omega_{\rm r}}\right| dt\right]^2, \tag{33}$$

where $|\bullet|$ stands for the operator norm (in general it is the maximal eigenvalue minus the minimal; for $H \propto \sigma_\theta$ it can be written in this way). For $\omega_{\rm r} = 0$:

$$\frac{\partial H}{\partial \omega_{\rm r}} = \left[-(A_1 - A_2)\, t \sin(\omega_{\rm s} t) + (B_1 - B_2)\, t \cos(\omega_{\rm s} t)\right]\sigma_Z. \tag{34}$$

Therefore given that $\omega_{\rm s} t \gg 1$, the maximal $I_{\rm r}$ reads:

$$I_{\rm r} = \left(\frac{2}{\pi}\right)^2 \left[(A_1 - A_2)^2 + (B_1 - B_2)^2\right] t^4. \tag{35}$$

Therefore given that A_i, B_i are i.i.d. with variance σ^2 an upper bound for the average $I_{\rm r}$ is:

$$I_{\rm r} \leq \frac{16}{\pi^2}\sigma^2 t^4. \tag{36}$$

Given A_i, B_i the control that achieves the optimal $I_{\rm r}$ consists of applying π-pulses whenever $\frac{\partial H}{\partial \omega_{\rm r}}$ flips a sign; therefore it requires knowing A_i, B_i, which is unrealistic in the setting described in the paper. In practice we need to apply the same control to every realization of A_i, B_i, hence this upper bound is not achievable. Using the method presented in the paper we obtain $I_{\rm r} = \frac{8\sigma^2 t^4}{\pi^4}$, hence lower by a factor of $2\pi^2$ from this upper bound. Whether our method is optimal given this noise model is left as an open question.

Supplementary Note VII. Multiparameter estimation of all the parameters

In an actual experimental scenario all the parameters are unknown, and since the estimation protocol of ω_r depends on ω_s (the pulses frequency should be detuned from ω_s by $\frac{2\pi}{\omega_s}$), a preliminary estimation of ω_s is necessary. We propose the following protocol: For a preliminary estimation of all the parameters $(\omega_r, \omega_s, \sigma)$ the traditional protocol is applied; namely vary the pulses frequency (denoted as ω_p) and make a large number of measurements for each ω_p. The next step is to fit the obtained data points (or make MLE analysis). This should provide a good estimation of ω_s and σ, the estimation of ω_r however will not be good enough (unless by chance we hit very close to the resonance points, and perform enough measurements in this point). The performance of this estimation method is illustrated in supplementary fig. 1.

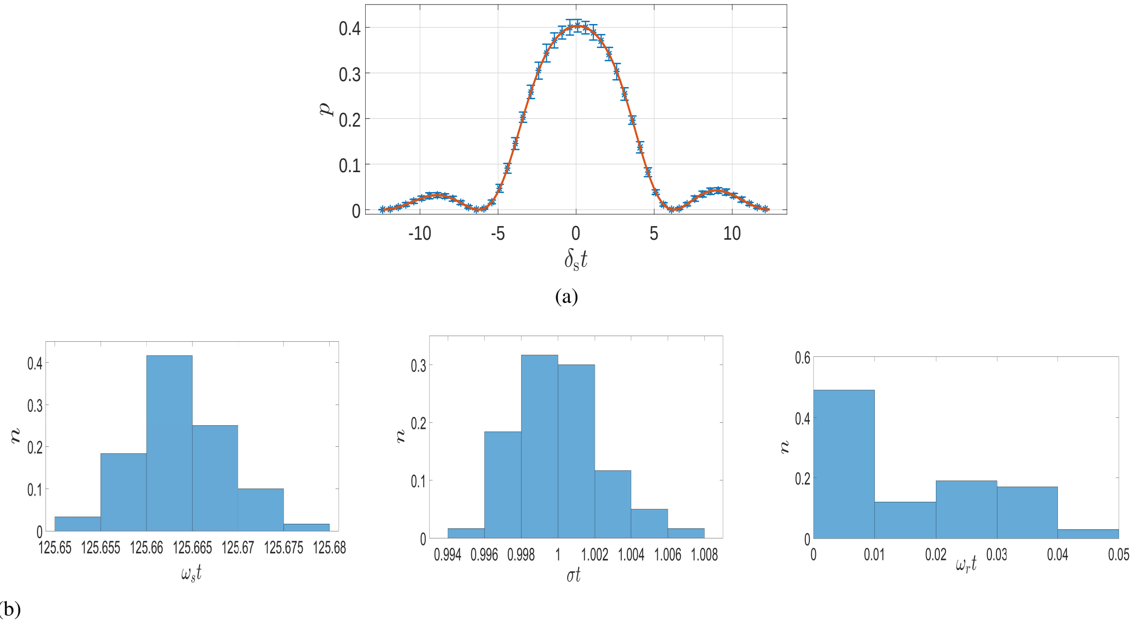

Supplementary Figure 1: Preliminary estimation of the parameters achieved by varying δ_s and fitting the probability as a function of δ_s. A numerical example is presented. The fit is shown in (a) and the estimation errors are presented in (b). While the ω_s can be estimated efficiently , the estimation of ω_r is not good enough for resolution. For this illustration: $\sigma t = 1$, $\omega_s t = 40\pi$ and the number of measurements in each detuning is $n = 2 \cdot 10^4$.

The next step is to use the estimated ω_s, σ to apply our method. One can treat ω_s, σ as known and estimate ω_r, however this will create a bias (we also want to progressively improve the estimation of ω_s, such that the detuning of the pulses will be more accurate). Note that we cannot get information about three different parameters by measuring copies of the same density matrix (even if measuring different observables, the FI matrix will be singular). Therefore at least three different measurements are required: one measurement with $\delta_s t = 2\pi$, the resonance condition for estimation of ω_r and two other measurements with the optimal detunings for estimating ω_s, σ. The FI about ω_s, σ as a function of δ_s is shown in supplementary fig. 2. Quite interestingly for both ω_s, σ the optimal $\delta_s \to \frac{2\pi}{t}$ as $\sigma \to \infty$ (this is because as σ becomes larger the exponential decay becomes stronger and one needs to get closer to $\delta_s t = 2\pi$).

The optimal FI about ω_s scales as $\sigma^2 t^4$ and is comparable to the optimal FI about ω_r. The optimal FI about σ behaves in an unusual manner: Usually the FI about the amplitude grows as t^2, while here the optimum (for $\sigma t > 1$) is $I_\sigma \sim \frac{0.63}{\sigma^2}$. It does not depend on t, and it drops as σ gets larger. This behavior is somewhat similar to sensing the standard deviation of the amplitude of a stationary signal ($H = A\sigma_Z$ where $A \sim N(0, \sigma)$).

Since we are dealing with a multivariable estimation, the Cramér-Rao bound is given by the Fisher information matrix in the following way [9]: $(\Delta x)^2 = \left(I^{-1}\right)_{x,x}$. We would like then to calculate the FI matrix. The full expression of the matrix is quite involved, however we can easily observe that as $\omega_r \to 0$ the FI matrix converges to a block diagonal matrix, with I_r as one of its eigenvalues. The FI matrix per three measurements is the sum of the FI matrices of each measurement: $I^{(1)} + I^{(2)} + I^{(3)}$. Therefore the FI matrix per single measurement reads: $I = \frac{1}{3}\left(I^{(1)} + I^{(2)} + I^{(3)}\right)$. Denoting the FI matrix that corresponds to

$\delta_s t = 2\pi$ as $I^{(1)}$, we observe that:

$$\frac{\left(\frac{\partial p}{\partial \omega_s}\right)^2}{p(1-p)}, \frac{\left(\frac{\partial p}{\partial \sigma}\right)^2}{p(1-p)}, \frac{\left(\frac{\partial p}{\partial \sigma}\right)\left(\frac{\partial p}{\partial \omega_r}\right)}{p(1-p)}, \frac{\left(\frac{\partial p}{\partial \omega_s}\right)\left(\frac{\partial p}{\partial \omega_r}\right)}{p(1-p)} \to 0 \Longrightarrow I^{(1)} = \begin{pmatrix} I_r & 0 & 0 \\ 0 & 0 & 0 \\ 0 & 0 & 0 \end{pmatrix} \tag{37}$$

Regarding $I^{(2)}, I^{(3)}$, note that for them (since $\delta_s t \neq 2\pi n$) we have that:

$$\frac{\left(\frac{\partial p}{\partial \omega_r}\right)^2}{p(1-p)}, \frac{\left(\frac{\partial p}{\partial \omega_r}\right)\left(\frac{\partial p}{\partial \omega_s}\right)}{p(1-p)}, \frac{\left(\frac{\partial p}{\partial \omega_r}\right)\left(\frac{\partial p}{\partial \sigma}\right)}{p(1-p)} \to 0 \Longrightarrow I^{(j)} = \begin{pmatrix} 0 & 0 & 0 \\ 0 & I_{2,2}^{(j)} & I_{2,3}^{(j)} \\ 0 & I_{3,2}^{(j)} & I_{3,3}^{(j)} \end{pmatrix}. \tag{38}$$

Therefore the FI matrix per single measurement reads:

$$I = \frac{1}{3}\begin{pmatrix} I_r & 0 & 0 \\ 0 & I_{2,2} & I_{2,3} \\ 0 & I_{3,2} & I_{3,3} \end{pmatrix}, \tag{39}$$

and thus $\Delta\omega_r = \sqrt{\frac{3}{I_r}}$, we therefore get an extra factor of $\sqrt{3}$ due to these chunks of three measurements. The standard deviation obtained in practice (with MLE) is a bit above the expected analytical values, as can be seen in supplementary fig. 3.

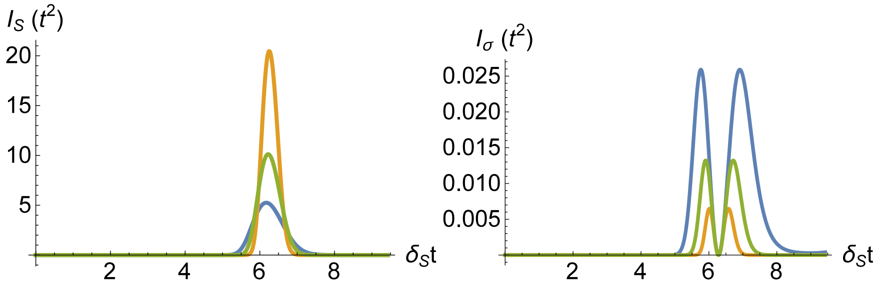

(a)

(b)

Supplementary Figure 2: (a) The FI about ω_s as function of $\delta_s t$ for different values of σ ($\sigma t = 5$: Orange, $\sigma t = 7$: Green, $\sigma t = 10$: Blue).
(b) The FI about σ as function of $\delta_s t$ for different values of σ (same colors). In both plots $\omega_r = 0$. Interestingly, as $\sigma t \to \infty$ the optimal $\delta_s t \to 2\pi$.

Supplementary Note VIII. Limitation due to incoherence

In this part we derive the effect of incoherence of the signal (during the measurement) on the method, where we consider a model in which the quadratures undergo identical and independent OU process. The OU process is defined as $dA_i = -\gamma A_i\, dt + \sigma_n\, dW_t^{A_i}$, and similarly for B_i. To get the effect, we need to calculate the transition probability $p = \langle \sin(\phi)^2 \rangle$, where ϕ is the accumulated phase. Note that:

$$\phi = \sum_{i=1}^{2} \int_0^T \left(A_i(t)\cos(\omega_i t) + B_i(t)\sin(\omega_i t)\right) dt, \tag{40}$$

where $A_i(t) = A_i(0)\exp(-\gamma t) + \sigma_n \int_0^t e^{-\gamma(t-s)} dW_s^{A_i}$, and the same holds for $B_i(t)$. Therefore ϕ can be written as the sum $\phi = \phi_{av} + \phi_n$, where:

$$\phi_{av} = \sum_{i=1}^{2} \int_0^T \left(A_i(0)\, e^{-\gamma t}\cos(\omega_i t) + B_i(0)\, e^{-\gamma t}\sin(\omega_i t)\right) dt, \tag{41}$$

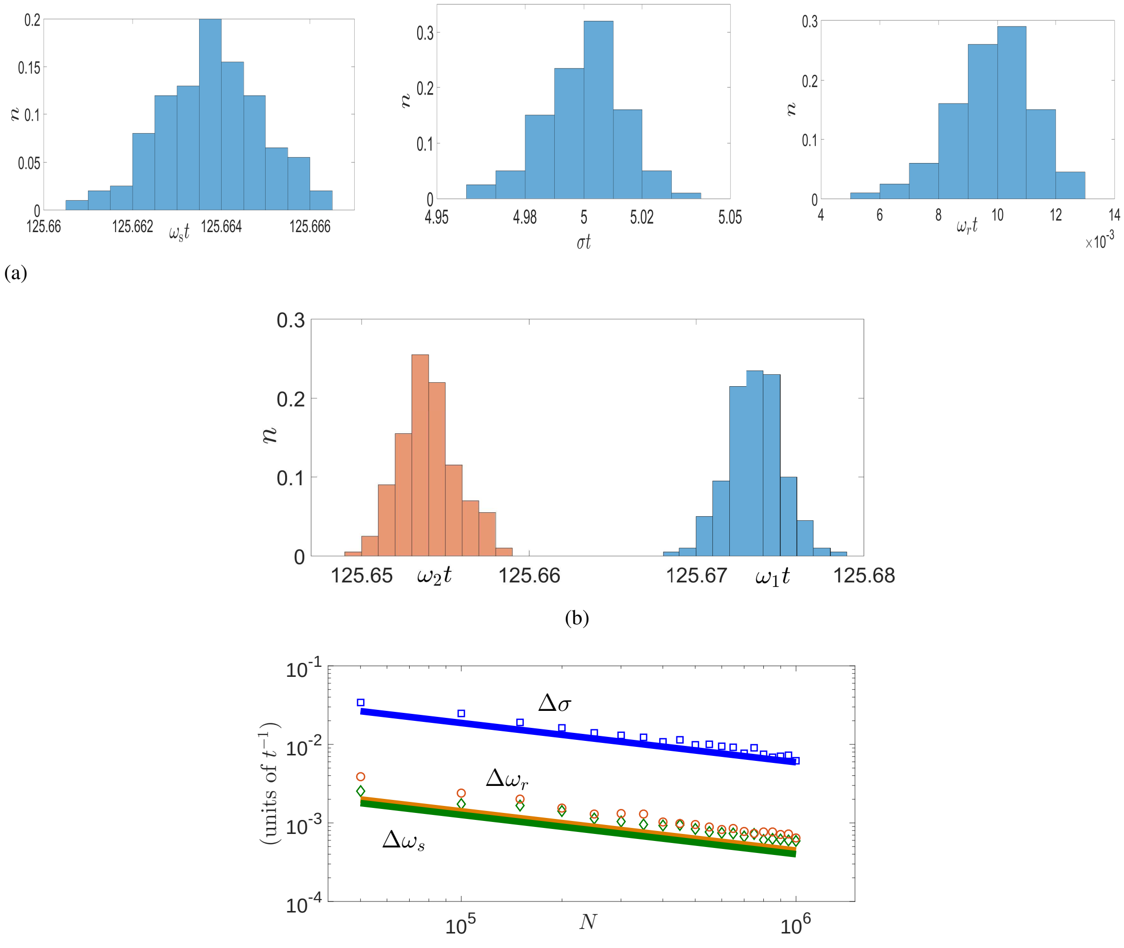

Supplementary Figure 3: The second stage of the protocol consists of measurements in three different detunings (each one is optimal for the estimation of a different parameter; one of them is therefore $\delta_s = \frac{2\pi}{t}$). In this example: $\delta_s t = 0.01$, $\sigma t = 5$ and the number of measurements in each detuning is $N = 3 \cdot 10^5$. The histograms for the different parameters are presented in (a). It can be seen that resolution is achieved as $\Delta\omega_\mathrm{r} = \frac{1}{7.5}\omega_\mathrm{r}$. In (b) the histograms of ω_1, ω_2 are presented, it can be seen that the frequencies are clearly resolved. (c) The RMSE (root mean square error) in estimating the parameters as a function of N. The solid lines are the theoretical expectations (green (bottom): $\Delta\omega_\mathrm{s}$, orange (middle): $\Delta\omega_\mathrm{r}$, blue (top): $\Delta\sigma$). The points represent the RMSE achieved in an actual maximum likelihood estimation (green (diamonds):$\Delta\omega_\mathrm{s}$, orange (circles): $\Delta\omega_\mathrm{r}$, blue (squares): $\Delta\sigma$). It can be seen that there is no divergence: The RMSE of all the parameters scale as $N^{-0.5}$. In fact $\Delta\omega_\mathrm{r}$ is very close to $\Delta\omega_\mathrm{s}$, whereas the worst is $\Delta\sigma$.

and:

$$\phi_n = \sum_{i=1}^{2} \sigma_n \int_0^T dt \left(\cos(\omega_i t) \int_0^t e^{-\gamma(t-s)} dW_s^{A_i} + \sin(\omega_i t) \int_0^t e^{-\gamma(t-s)} dW_s^{B_i} \right). \tag{42}$$

Observe that ϕ_n is a Gaussian random variable with $\langle \phi_n \rangle = 0$, and:

$$\langle \phi_n^2 \rangle = \sum_{i=1}^{2} \sigma_n^2 \int_0^T ds \left[\int_s^T \cos(\omega_i t) e^{-\gamma(t-s)} dt \right]^2 + \sum_i \sigma_n^2 \int_0^T ds \left[\int_s^T \sin(\omega_i t) e^{-\gamma(t-s)} dt \right]^2 \tag{43}$$

We would like now to take $\omega_s T = 2\pi$ in the regime of: $\gamma T \ll 1$, $\phi_n \ll 1$, $\omega_r T \ll 1$. In this case $p = \langle \sin(\phi)^2 \rangle \approx \langle \phi_{av}^2 \rangle + \langle \phi_n^2 \rangle$. In leading orders:

$$\langle \phi_n^2 \rangle \approx \sigma_n^2 T^3 \left(\frac{1}{\pi^2} + O(\gamma T) \right), \tag{44}$$

and:

$$\begin{aligned} \langle \phi_{av}^2 \rangle &\approx \langle \left[\frac{(A_1(0) - A_2(0))}{2\pi} \omega_r T^2 + \sum_{i=1}^{2} B_i(0) \frac{\gamma T^2}{2\pi} \right]^2 \rangle = \\ &= \sum_{i=1}^{2} \langle A_i(0)^2 \rangle \frac{1}{4\pi^2} \omega_r^2 T^4 + \sum_{i=1}^{2} \langle B_i(0)^2 \rangle \frac{1}{4\pi^2} \gamma^2 T^4 = \\ &= \frac{\sigma_n^2}{4\pi^2 \gamma} \omega_r^2 T^4 + \frac{\sigma_n^2}{4\pi^2} \gamma T^4. \end{aligned} \tag{45}$$

The second term ($\sum_i \frac{\sigma_n^2}{4\pi^2} \gamma T^4$) can be neglected as it is much smaller than $\langle \phi_n^2 \rangle \sim \sigma_n^2 T^3$. Therefore:

$$p \approx \langle \phi_{av}^2 \rangle + \langle \phi_n^2 \rangle \approx \frac{\sigma_n^2 T^3}{\pi^2} + \frac{\sigma_n^2}{4\pi^2 \gamma} \omega_r^2 T^4, \tag{46}$$

using the notation in the main text $\frac{\sigma_n^2 T^3}{\pi^2} = n$, and the resolution condition is thus $\omega_r^2 \frac{T}{\gamma} \gg 1$.

Supplementary Note IX. Limitation due to dephasing of the probe

Let us now find the implications of noise inflicted on the probe: specifically we consider a Markovian dephasing. Intuitively this should set an additional limitation: the transition probability does not vanish now due to two reasons: the finite ω_r, namely the term $(\sigma t)^2 (\omega_r t)^2$, and also due to a dephasing rate of κ, namely a term of κt. Hence resolution can be achieved if the first term is larger than the second, hence the condition is $\frac{\omega_r \sigma}{(\kappa^2)} \gg 1$.

In more detail, given our Hamiltonian $H = f(t)\sigma_z$ and a dephasing rate κ, the time evolution is given by the Master equation:

$$\frac{d\rho}{dt} = -i[f(t)\sigma_z, \rho] + \kappa(\sigma_z \rho \sigma_z - \rho). \tag{47}$$

Initializing and measuring in σ_x basis, we get the transition probability $p = 0.5 - 0.5e^{-2\kappa t}\cos(2\phi)$ (where ϕ is the accumulated phase). Averaging over the different realizations we get: $p = 0.5\left(1 - \exp\left(-\frac{(\sigma t)^2 (\omega_r t)^2}{\pi^2} - 2\kappa t\right)\right)$, and the FI reads:

$$I_r = \frac{4\omega_r^2 \sigma^4 t^8}{\pi^4 \left[\left(\exp\left(4\kappa t + 2\omega_r^2 \sigma^2 t^4 / \pi^2\right) - 1 \right) \right]}. \tag{48}$$

Hence the noiseless FI is retrieved only for $\frac{\omega_r \sigma}{(\kappa^2)} \gg 1$. Note that the effect of dephasing in this problem is quite different: first, the FI is not necessarily close to the noiseless FI for $\kappa t \ll 1$, the condition $\frac{\omega_r \sigma}{(\kappa^2)} \gg 1$ must hold. Second, since there is a competition between $(\sigma t)^2 (\omega_r t)^2$ and κt, and for short enough times the second term is always larger, then the noiseless FI is retrieved only after a minimal time (goes as $\sim \frac{\kappa^{1/3}}{\sigma^{2/3} \omega_r^{2/3}}$) . This behavior is illustrated in supplementary fig. 4.

We therefore observe that Markovian noise on the qubit (such as dephasing) imposes a resolution limit, this invokes a natural question: can error correction protocols remove these limitations imposed by Markovian noise? Note that this question is not analogous to the achievability of Heisenberg scaling which was addressed in [10–12]. The prospects for quantum error correction in these resolution problems are left for future work, however we point out that this limit can be eliminated provided that errors can be detected, since one does not need to correct the errors. Given that error detection is possible, we can postselect the measurements without error and perform estimation according to them. These errors thus reduce the precision, but they do not impose a limitation. This implies that the error correction condition to remove limitation in this problem should be different from the error correction condition for Heisenberg scaling. Let us denote the jump operators in the Master equation as $\{L_j\}_j$.

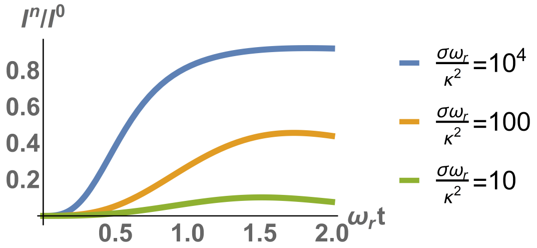

Supplementary Figure 4: The FI with dephasing (I^n) divided by the noiseless FI (I^0), for different values of $\frac{\sigma\omega_\mathrm{r}}{\kappa^2}$. Note that as $\frac{\sigma\omega_\mathrm{r}}{\kappa^2}$ gets higher the noisy FI can get closer to the noiseless FI, however there is a minimal time from which this can be achieved. This minimal time goes as $\sim \frac{\kappa^{1/3}}{\sigma^{2/3}\omega_\mathrm{r}^{2/3}}$.

For Heisenberg scaling, the error correction condition is $H \notin \mathrm{span}\left\{I, L_j, L_j^\dagger, L_i^\dagger L_j\right\}_{i,,j}$ [10–12], since we need both to detect and to correct. In this case, since we need only to detect, the error correction condition should be: $H \notin \mathrm{span}\left\{I, L_j^\dagger, L_j\right\}_j$. This implies, for example, that if the only noise source is amplitude damping (namely the only jump operator is σ_-), then detection is possible, and we can overcome the limitation.

Supplementary Note X. Superresolution with QFT

Consider the Hamiltonian in supplementary eq. 18, and assume a relatively long coherence time (in which phases and amplitudes are constant), such that sampling is performed. Namely Ramsey measurements are performed in different times, as it is described in [4, 13, 14]. The length of each measurement is τ and the total sampling time is $T = N\tau$. The standard way to analyze this data is to perform a Fourier transform and fit the power spectrum. However this method suffers from a resolution limit [15], since the probability is symmetric with respect to ω_r and the noise does not vanish.

We claim that storing the data in a quantum state (using memory qubits) and using the same trick of nullifying the projection noise, then resolution limit can be beaten. In a standard Ramsey experiment the state of the probe, after phase accumulation, is $\frac{1}{\sqrt{2}}\left(|0\rangle + e^{i\phi}|1\rangle\right)$. If we entangle the probe to memory qubits in each phase acquisition, the following state of the memory qubits can be generated:

$$|\psi\rangle = \frac{1}{\sqrt{N}}\left(\sum_{j=0}^{N-1}|j\rangle e^{i\phi_j}\right), \tag{49}$$

where $\phi_j = \tau\left[\sum_i A_i \cos(\omega_i t_j) + B_i \sin(\omega_i t_j)\right]$, $(t_j = j\tau)$. The idea is that for $\omega_\mathrm{r} = 0$ only harmonics of ω_s can be measured, and the probability to measure the other frequencies goes as ω_r^2. To see this note that

$$\phi_j(\omega_\mathrm{r} = 0) = \tau\left[\sum_i A_i \cos(\omega_\mathrm{s} t_j) + B_i \sin(\omega_\mathrm{s} t_j)\right] = \Omega \sin(\omega_\mathrm{s} t_j + \varphi), \tag{50}$$

and therefore:

$$e^{i\phi_j(\omega_\mathrm{r}=0)} = \sum_{k=-\infty}^{\infty} J_k(\Omega)\exp(ik\varphi)\exp(ik\omega_\mathrm{s} j\tau), \tag{51}$$

where this expansion to harmonics of ω_s is the Jacobi-Anger expansion.

Since we want to make sure that ω_s (and integer multiples of it) will be included in the Fourier basis, we need to set $T = \frac{2\pi}{\omega_\mathrm{s}} m$ (integer m). In order to avoid too many harmonics, we also set $\tau = \frac{2\pi}{\omega_\mathrm{s}}\frac{1}{n}$ (integer n).

It is now simple to see that with this choice, the only frequencies that can be measured in QFT are: $0, \omega_\mathrm{s}, \ldots, (n-1)\,\omega_\mathrm{s}$, as the state reads:

$$|\psi_0\rangle = \sum_{l=0}^{n-1} a_l |l\omega_\mathrm{s}\rangle, \tag{52}$$

where $a_l = \sum_{k=-\infty}^{\infty} J_{nk+l}(\Omega) \exp(i(nk+l)\varphi)$.

So for example given a noise model of a random phase, the density matrix is diagonal in the Fourier basis: $\rho = \sum_{l=0}^{n-1} p_l |l\omega_{\rm s}\rangle\langle l\omega_{\rm s}|$, where $p_l = \sum_{k=-\infty}^{\infty} |J_{nk+l}(\Omega)|^2$, hence the optimal measurement basis is the Fourier basis (and it is enough to measure whether we get harmonics of $\omega_{\rm s}$ or not).

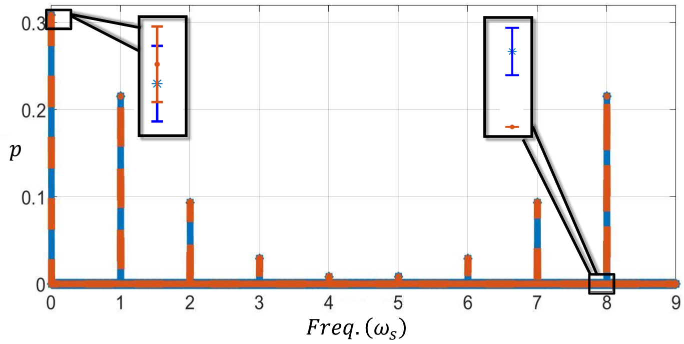

Supplementary Figure 5: Illustration of superresolution with QFT: probabilities of the different frequencies for $\omega_{\rm r} = 0$ (dashed, orange lines) and $\omega_{\rm r} T = 0.05$ (solid, blue line). The probabilities to measure frequencies that are not harmonics of $\omega_{\rm s}$ goes as $\omega_{\rm r}^2$, and thus superresolution can be achieved by measuring these frequencies. In this illustration, the quadratures are random with $\sigma\tau = 1$, and the number of repetitions is 10^5.

Let us now find the probability to measure frequencies that are not harmonics of $\omega_{\rm s}$, for $\omega_{\rm r} T \ll 1$. We denote the projector on the other frequencies (not harmonics of $\omega_{\rm s}$) as Π, so we are interested in finding $p = |\Pi|\psi\rangle|^2$. Of course: $|\psi\rangle = |\psi_0\rangle + \omega_{\rm r}\frac{d|\psi\rangle}{d\omega_{\rm r}} + \mathcal{O}\left(\omega_{\rm r}^2\right)$, and since $\Pi|\psi_0\rangle = 0$, we get that: $p \approx \omega_{\rm r}^2 |\Pi \frac{d|\psi\rangle}{d\omega_{\rm r}}|^2$. Now:

$$\frac{d|\psi\rangle}{d\omega_{\rm r}} = \sum_{j=1}^{N} \exp(i\phi_j(\omega_{\rm r}=0))(i\omega_{\rm r} t_j)\left[(B_1 - B_2)\tau\cos(\omega_{\rm s} t_j) + (A_2 - A_1)\tau\sin(\omega_{\rm s} t_j)\right]|j\rangle,$$

note that we can expand:

$$\exp(i\phi_j(\omega_{\rm r}=0))\left[(B_1 - B_2)\tau\cos(\omega_{\rm s} t_j) + (A_2 - A_1)\tau\sin(\omega_{\rm s} t_j)\right] = \sum_{l=0}^{n-1} b_l \exp(il\omega_{\rm s} t_j), \tag{53}$$

Therefore:

$$\frac{d|\psi\rangle}{d\omega_{\rm r}} = \frac{i}{\sqrt{N}} \sum_{l=0}^{n-1} b_l \sum_{j=0}^{N-1} t_j \exp(il\omega_{\rm s} t_j)|j\rangle. \tag{54}$$

For convenience let us denote $|r_k\rangle = \frac{1}{\sqrt{N}} \sum_{j=1}^{N} t_j \exp(ik\omega_{\rm s} t_j)|j\rangle$, then with this notation:

$$\frac{d|\psi\rangle}{d\omega_{\rm r}} = i\sum_{l=0}^{n-1} b_l |r_l\rangle. \tag{55}$$

Now given that $T \gg \frac{2\pi}{\omega_{\rm s}}$, the state $|r_l\rangle$ will have a non-negligible overlap only with frequencies close enough to $|l\omega_{\rm s}\rangle$, this leads us to make two approximations: $i \neq k \Rightarrow \langle r_k|\Pi|r_i\rangle = 0$ (different $|r_k\rangle$'s overlap orthogonal frequencies) and $|\Pi|r_k\rangle|^2 = \langle r_k|r_k\rangle - |\langle k\omega_{\rm s}|r_k\rangle|^2$ (the only harmonic that overlaps $|r_k\rangle$ is $|k\omega_{\rm s}\rangle$).

Due to the first approximation:

$$p \approx \omega_{\rm r}^2 |\Pi \frac{d|\psi\rangle}{d\omega_{\rm r}}|^2 \approx \omega_{\rm r}^2 \sum_{l=0}^{n-1} |b_l|^2 |\Pi|r_l\rangle|^2. \tag{56}$$

observe now that:

$$\begin{aligned}\langle r_k | r_k \rangle &= \frac{1}{N}\sum_j t_j^2 \approx \frac{T^2}{3} \\ \langle k\omega_{\mathrm{s}} | r_k \rangle &= \frac{1}{N}\sum_j t_j \approx \frac{T}{2}.\end{aligned} \tag{57}$$

Then due to the second approximation:

$$p = \frac{1}{12}\omega_{\mathrm{r}}^2 T^2 \sum_{l=0}^{(n-1)} |b_l|^2. \tag{58}$$

It is now simple to see that: $\sum_{l=0}^{(n-1)} |b_l|^2 = \tau^2 \frac{1}{2}\left[(A_1 - A_2)^2 + (B_1 - B_2)^2\right]$. Therefore taking the model of random quadratures (each with variance σ^2), we get: $p = \frac{1}{6}\omega_{\mathrm{r}}^2 T^2 (\sigma\tau)^2$.

Supplementary Note XI. Different noise models

We showed in the main text that given the following effective Hamiltonian (this already takes into account the pulses, so all the relevant factors have been absorbed into the amplitudes):

$$H = [A_1 \cos(\delta_1 t) + B_1 \sin(\delta_1 t) + A_2 \cos(\delta_2 t) + B_2 \sin(\delta_2 t)]\,\sigma_z, \tag{59}$$

and a certain noise model of the amplitudes, the FI is calculated according to the average transition probability:

$$p = \int \sin^2(\phi) \prod_i p(A_i)\, p(B_i)\, dA_i\, dB_i. \tag{60}$$

Using the control method proposed in this paper (applying π pulses such that $\delta_s t = 2\pi$), we obtain that $\phi \approx \frac{(A_1 - A_2)}{2\pi}\omega_{\mathrm{r}} t^2 \rightarrow p_a \propto \omega_{\mathrm{r}}^2$. Therefore a non vanishing I_{r} is achieved as long as $\int (A_1 - A_2)^2 p(\mathbf{A},\mathbf{B})\, d\mathbf{A}\, d\mathbf{B} \neq 0$. Since our primary interest is in NMR we assumed the noise model relevant to unpolarized NMR in which A_i, B_i are Gaussian i.i.d. with a distribution of $N(0,\sigma)$. Assuming this noise model, the average transition probability,p, is given by:

$$p = 0.5\left(1 - \exp\left(-8\sum_i \frac{\sigma^2}{\delta_i^2}\sin^2\left(\frac{\delta_i t}{2}\right)\right)\right), \tag{61}$$

For $\delta_s t = 2\pi$, $p_a \approx \frac{2\sigma^2}{\omega_{\mathrm{s}}^2}\omega_{\mathrm{r}}^2 t^2$, and thus $I_{\mathrm{r}} = \frac{2\sigma^2 t^4}{\pi^2}$.

For classical signals (such as microwave signals generated by AC wires) a different noise model should be taken into account. For these signals the amplitude of the field ($\sqrt{A^2 + B^2}$) is constant, while the phase ($\arctan\left(\frac{B}{A}\right)$) distributes uniformly. It is easy to verify that in this case $\int (A_1 - A_2)^2 p(\mathbf{A},\mathbf{B})\, d\mathbf{A}\, d\mathbf{B} \neq 0$, and thus a finite FI is achieved. A detailed analysis shows that:

$$p = \frac{1}{2}\left(1 - J_0\left(\frac{4\Omega_1}{\delta_1}\sin\left(\frac{\delta_1 t}{2}\right)\right) J_0\left(\frac{4\Omega_2}{\delta_2}\sin\left(\frac{\delta_2 t}{2}\right)\right)\right), \tag{62}$$

where $\Omega_i = \sqrt{A_i^2 + B_i^2}$, and these amplitudes are constants and identical. Taking $\delta_s t = 2\pi$, we get:

$$p \approx \frac{(\Omega t)^2}{(2\pi)^2}\omega_{\mathrm{r}}^2 t^2 \Rightarrow I = \frac{4\Omega^2}{(2\pi)^2} t^4. \tag{63}$$

Supplementary References

[1] Braunstein, S. L. & Caves, C. M. Statistical distance and the geometry of quantum states. *Physical Review Letters* **72**, 3439 (1994).

[2] Tsang, M., Nair, R. & Lu, X.-M. Quantum theory of superresolution for two incoherent optical point sources. *Physical Review X* **6**, 031033 (2016).

[3] Pang, S. & Jordan, A. N. Optimal adaptive control for quantum metrology with time-dependent hamiltonians. *Nature communications* **8**, 14695 (2017).

[4] Schmitt, S. *et al.* Submillihertz magnetic spectroscopy performed with a nanoscale quantum sensor. *Science* **356**, 832–837 (2017).

[5] Jordan, A. N. Classical-quantum sensors keep better time. *Science* **356**, 802–803 (2017).

[6] Yang, J., Pang, S. & Jordan, A. N. Quantum parameter estimation with the landau-zener transition. *Physical Review A* **96**, 020301 (2017).

[7] Gefen, T., Jelezko, F. & Retzker, A. Control methods for improved fisher information with quantum sensing. *Physical Review A* **96**, 032310 (2017).

[8] Naghiloo, M., Jordan, A. & Murch, K. Achieving optimal quantum acceleration of frequency estimation using adaptive coherent control. *Physical review letters* **119**, 180801 (2017).

[9] Cramér, H. *Mathematical methods of statistics (PMS-9)*, vol. 9 (Princeton university press, 2016).

[10] Sekatski, P., Skotiniotis, M., Kołodyński, J. & Dür, W. Quantum metrology with full and fast quantum control. *Quantum* **1**, 27 (2017).

[11] Zhou, S., Zhang, M., Preskill, J. & Jiang, L. Achieving the heisenberg limit in quantum metrology using quantum error correction. *Nature communications* **9**, 78 (2018).

[12] Demkowicz-Dobrzański, R., Czajkowski, J. & Sekatski, P. Adaptive quantum metrology under general markovian noise. *Physical Review X* **7**, 041009 (2017).

[13] Boss, J., Cujia, K., Zopes, J. & Degen, C. Quantum sensing with arbitrary frequency resolution. *Science* **356**, 837–840 (2017).

[14] Glenn, D. R. *et al.* High-resolution magnetic resonance spectroscopy using a solid-state spin sensor. *Nature* **555**, 351 (2018).

[15] Rotem, A. *et al.* Limits on spectral resolution measurements by quantum probes. *Phys. Rev. Lett.* **122**, 060503 (2019). URL https://link.aps.org/doi/10.1103/PhysRevLett.122.060503.
